# Comparative study on the effect of different high-frequency chest wall oscillation and back patting modes on sputum excretion in patients with severe pneumonia

**DOI:** 10.3389/fmed.2025.1558861

**Published:** 2025-05-27

**Authors:** Bing Tang, Bin Gao, Li Tang, Yunying Li, Xiaomin Xiong, Boyuan Wang, Zhe Xu, Gexin Xiao, Zunxiong Xiao

**Affiliations:** ^1^Guangyuan Central Hospital, Guangyuan, China; ^2^Beijing Xiaotangshan Hospital, Beijing, China; ^3^Department of Biomedical Sciences, City University of Hong Kong, Hong Kong, Hong Kong SAR, China; ^4^National Institute of Hospital Administration (NIHA), Beijing, China; ^5^The First Affiliated Hospital of Hunan University of Medicine, Huaihua, China; ^6^Hunan University of Medicine, Huaihua, China

**Keywords:** severe pneumonia, HFCWO, lung symptom, mechanical ventilation, sputum expulsion

## Abstract

**Background:**

The effect of different backslapping parameters on sputum discharge in SP patients is unclear.

**Objective:**

To investigate the effects of different back-patting modes on sputum clearance from the lungs of SP patients. To provide a theoretical basis for future clinical care and development of critical care robots.

**Methods:**

The patients with severe pneumonia in ICU of Guangyuan tertiary hospital were selected as the study object, and according to the specific conditions of the patients, different back-patting methods were selected and given different back-patting intensity, back-patting frequency, back-patting times, back-patting times, back-patting times, and back-patting times, respectively, to compare and analyse the effects of the different back-patting methods on the patient’s expectoration effect, blood oxygen concentration, and lung signs.

**Results:**

Finally, 143 patients with severe pneumonia were included. The volume of sputum expectoration, sputum viscosity, and blood oxygen saturation were significantly higher with different back patting patterns (*p* < 0.05), and the effective rate of treatment of pulmonary signs was significantly higher (*p* < 0.05).

**Conclusion:**

Specific high-frequency chest wall shock back-patterning can effectively improve sputum expectoration in SP patients, helping to restore lung function and reduce hospital stay.

## 1 Introduction

Severe pneumonia (SP) is a serious respiratory disease characterized by multisystem involvement including cardiac, renal and hepatic systems, with rapid progression and high mortality rates (30–50% in mechanically ventilated patients) (1, 2). Prolonged mechanical ventilation induces mucus hypersecretion and impaired mucociliary clearance, leading to retained secretions that exacerbate hypoxemia (3, 4). High-frequency chest wall oscillation (HFCWO) enhances mucus transport through two-phase gas–liquid interaction (5), showing immediate improvement in secretion clearance in ventilated patients (6), though its superiority over conventional airway clearance techniques remains controversial (7).

HFCWO has been shown to be effective in facilitating airway clearance in patients with cystic fibrosis (CF) and those on prolonged mechanical ventilation, with comparable results to traditional methods (8). A randomized controlled trial found that HFCWO can effectively improve lung function, alleviate respiratory distress and cough symptoms in patients with COPD and bronchiectasis, and has good safety (9). Other studies have found that HFCWO and traditional methods such as postural drainage and percussion have shown similar efficacy in improving lung function, with no significant difference in results (9, 10). Although HFCWO has demonstrated its effectiveness, there is a lack of sufficient evidence to assert the superiority of HFCWO over other methods in airway clearance techniques, and therefore, further research is necessary (6, 8, 11, 12).

A systematic review and meta-analysis (13) evaluated the comparative efficacy, safety, and patient compliance of high-frequency chest wall oscillation versus traditional chest physiotherapy (including postural drainage and manual percussion) in cystic fibrosis (CF) management. This study included seven randomized controlled trials with 542 CF patients to evaluate the effects of two therapies on lung function (such as FEV_1_), quality of life, mucus clearance efficacy, hospitalization rate, and side effects. The study indicated that the HFCWO group improved FEV_1_ by 8.2% (95% CI 5.1–11.3%, *p* ≤ 0.001) compared to traditional percussion-based airway clearance and increased sputum clearance volume by 25% (*p* = 0.02). However, the study has certain limitations, including high heterogeneity among the included studies and small sample sizes in some trials, warranting further expansion of sample sizes in future research.

Randomized controlled trial (14) evaluated the efficacy of HFCWO in patients with acute exacerbations of chronic obstructive pulmonary disease (COPD), including its impact on symptom relief, lung function improvement, hospital length of stay, and readmission rates. The study enrolled a total of 120 patients and followed them for 4 weeks. The authors reported that the HFCWO group demonstrated significant superiority over the conventional treatment group in improving FEV₁ and reducing hospitalization duration, with HFCWO shortening hospital stays by 2.1 days (conventional group: 7.3 days vs. HFCWO group: 5.2 days, *p* = 0.01) and improving FEV₁ by 15% (*p* ≤ 0.05). Although HFCWO was well-tolerated by patients, some reported mild chest discomfort. However, the study had limitations, including a small sample size and a lack of long-term follow-up data. Another randomized controlled study (15) evaluated the short-term mucus clearance efficacy and patient preferences of high-frequency chest wall oscillation (HFCWO) and traditional mucus clearance techniques in patients with chronic obstructive pulmonary disease (COPD). This study is divided into the HFCWO group, which uses HFCWO equipment (such as Vest daily) perform a 20 min treatment, as well as the traditional group, using ACBT or manual percussion position drainage for 20 min daily. A total of 30 patients were included in the study, which found that HFCC increased sputum clearance by 35% (*p* = 0.004) and SpO_2_ by 2.5% (*p* = 0.02). It is believed that HFCWO significantly increases sputum clearance within 24 h compared to the traditional treatment group, and patients are more inclined to choose HFCWO due to its convenience and comfort in operation.

A study investigated (16) the effects of high-frequency chest wall oscillation on respiratory function, mucus clearance capacity, and frequency of respiratory infections in patients with Duchenne muscular dystrophy (DMD). The research enrolled 50 DMD patients, with the intervention group receiving 20 min of daily HFCWO therapy over a period of 6 months. The authors suggested that the HFCWO group demonstrated superior outcomes compared to the conventional care group in reducing the frequency of respiratory infections and slowing the decline in pulmonary function [manifested by a decreased annual rate of forced vital capacity (FVC) decline]. The effectiveness of high-frequency chest wall oscillation (HFCWO) versus conventional breathing exercises in the management of postoperative atelectasis was compared in a randomized controlled trial. When 80 patients were enrolled, the HFCWO group showed an 85% resolution of atelectasis versus 60% in the conventional group (*p* = 0.02). In addition, ICU duration was reduced by 1.8 days in the HFCWO group (*p* = 0.03), and the duration of pulmonary re-expansion after surgery was significantly shorter than that in the control group. However, there was no statistically significant difference in the death rate of 30 days between the two groups (17).

**Figure 1:**
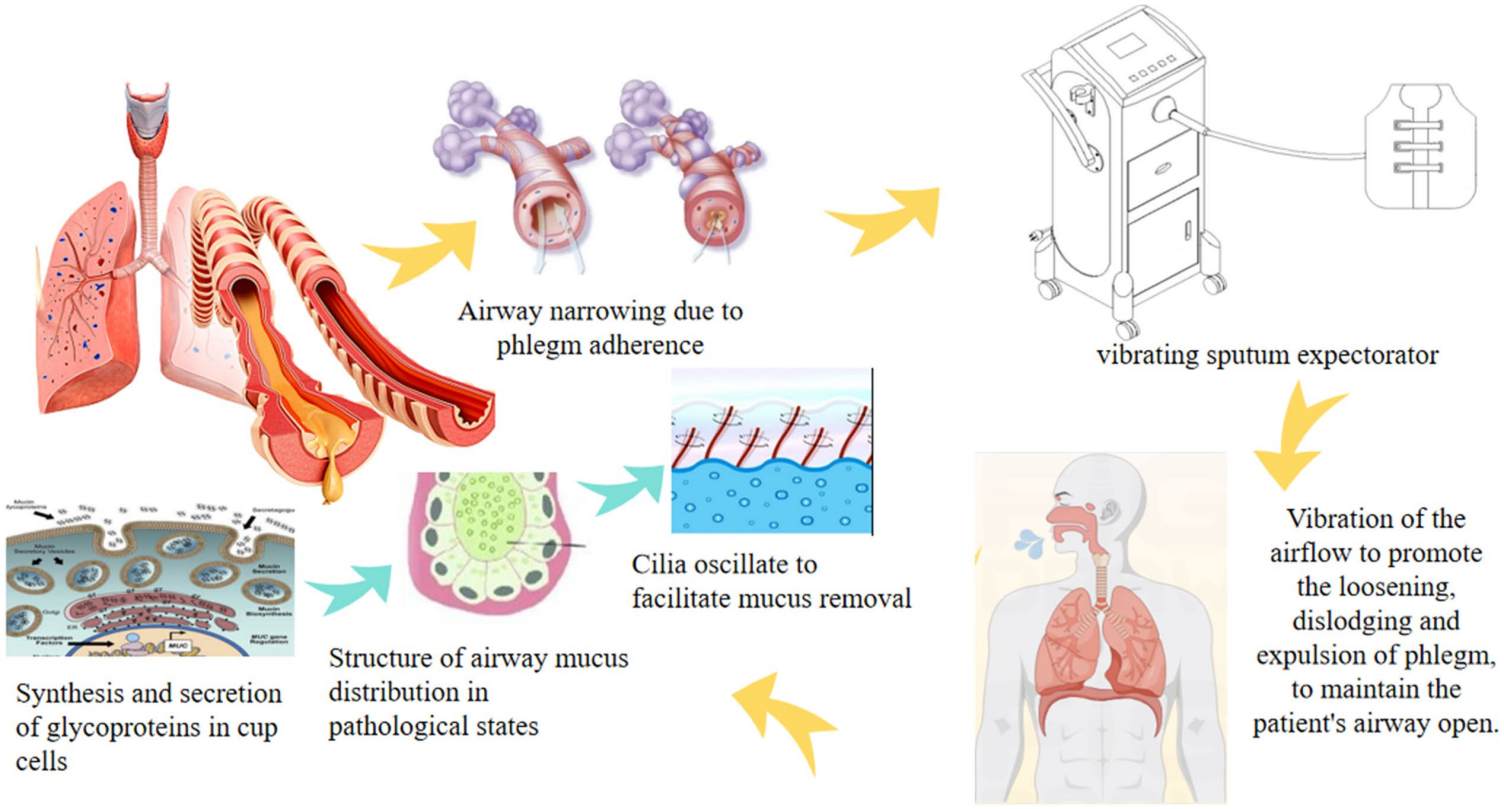
Principle of high-frequency chest wall oscillation sputum drainage system.

It is proposed to compare and analyse the effects of different frequencies, back strengths and back times on patients’ sputum clearance, to analyse their advantages and disadvantages, and to screen out the optimal back patting methods and treatment plans. This will provide a scientific basis for clinical application (Figure 1).

## 2 Materials and methods

In this study, 143 critically ill patients admitted during their hospitalization in a tertiary general hospital in Guangyuan City were selected as study subjects by convenience sampling method.

### 2.1 Patients

**Figure 2:**
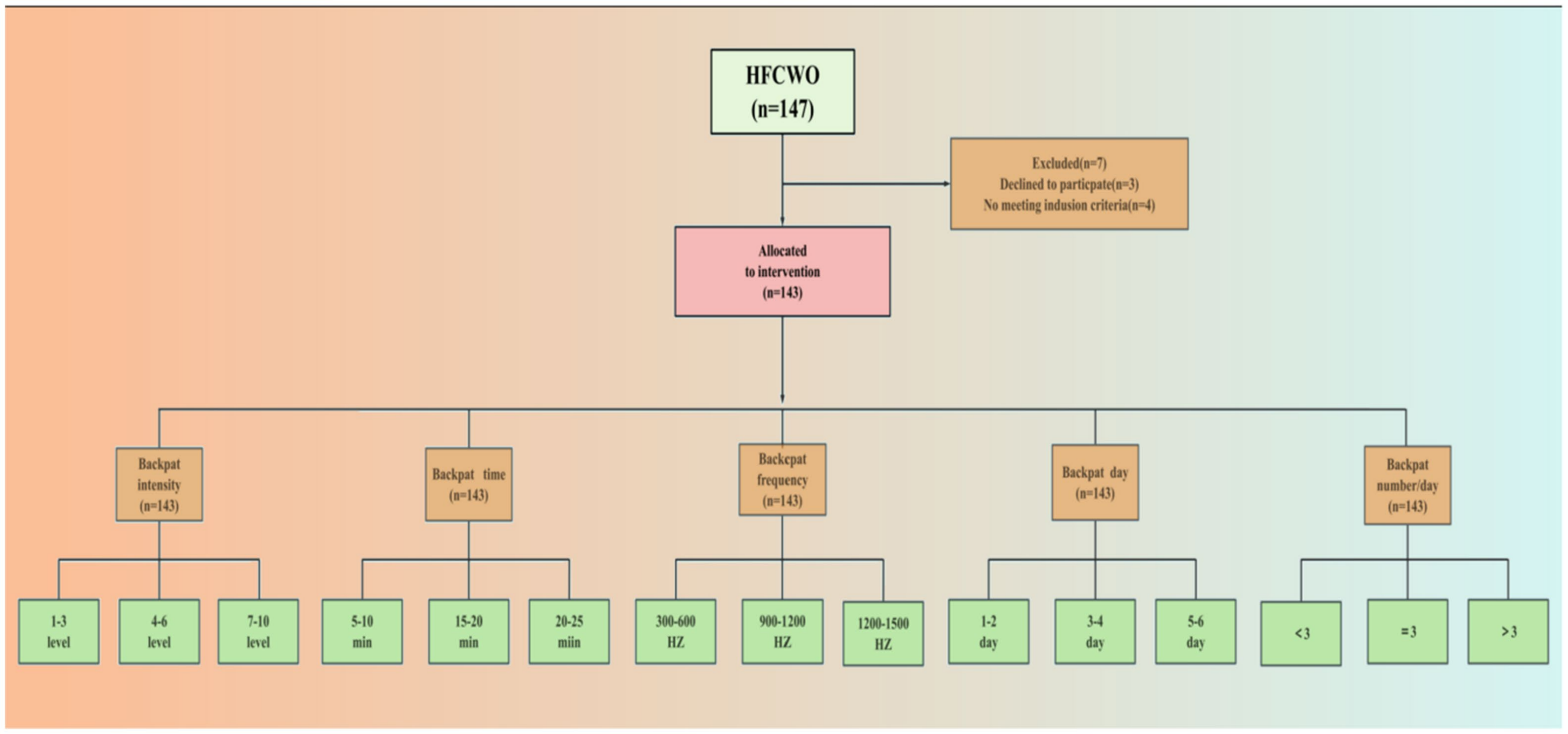
Flowchart of patient eligibility. HFCWO, high frequency chest wall oscillation.

A flowchart of the study is shown in Figure 2. Inclusion criteria of this study were as follows: (1) The diagnosis was established in accordance with the diagnostic criteria for severe pneumonia in Internal Medicine (9th edition), and the diagnosis was confirmed by lung bedside film, blood tests, and a combination of clinical symptoms. (2) Vital signs were stable and conscious. (3) Received ventilator therapy. (4) No psycho-behavioral disorders such as dementia or cognitive dysfunction. (5) Patient and family members were aware of and signed an informed consent for the study and were willing to participate in the trial. The elimination criteria were as follows: (1) Patients with malignant tumors of the lungs. (2) Patients who cannot be taken off the ventilator due to circulatory or respiratory failure or cerebrovascular accidents. (3) Patients with congenital bronchial developmental anomalies, coagulation disorders, etc. (4) Presence of foreign bodies in the airway. (5) Active bleeding, abnormal coagulation mechanisms, spinal fractures, inability to tolerate vibration, and comatose patients. (6) Presence of organic pathologies, and immunocompromised patients.

### 2.2 HFCWO support therapy

According to the inclusion and exclusion criteria, eligible patients were treated as follows. Patients were positioned in a 30° semi-Fowler posture with real-time respiratory phase monitoring. According to the patient’s body type, an appropriately sized vest (for BMI ≥25) or chest strap (for BMI ≤25) was selected to cover the entire thorax. Vest elasticity was adjusted to allow 2-finger insertion (≈20–30 N/cm^2^ compression force) and dynamically adapted to respiratory amplitude using a pneumatic feedback system. The operating mode was manually selected with pressure gradually increased from 1 to 12 steps (5–60 N), calibrated via embedded force transducers (FSR400, Interlink Electronics). Vibration frequency (2–25 Hz) was titrated based on patient tolerance, assessed by both subjective VAS scores and objective diaphragmatic electromyography. Each session duration (3–25 min) and daily patting count (1–5) were adjusted to maintain SpO₂ ≥92% and respiratory rate <25 breaths/min.

### 2.3 End points

The primary endpoints were sputum volume (mL) and sputum viscosity grading within 24 h of the intervention. Grade 1: sputum is like rice soup or foam, and no sputum is retained on the inner wall of the glass connector after suction. Grade 2: sputum is more viscous in appearance than Grade 1, and a small amount of sputum is retained on the inner wall of the glass connector after suction, but it is easily washed away. Grade 3: sputum is obviously viscous in appearance and is yellow in color, and the suction tube is often collapsed due to excessive negative pressure, and a large amount of sputum is retained on the inner wall of the glass connector, and it is not easy to wash away by water.

Secondary endpoints were (1) Oxygen saturation: after the completion of all high-frequency chest wall shock back-patting, 1–2 mL of arterial blood was collected under the patient’s comfortable and stable condition, and the arterial oxygen saturation of the two groups was determined using the blood gas analyser. (2) Pulmonary signs: the improvement of pulmonary signs such as cough, shortness of breath and wet rales in the lungs was observed in the patients after the intervention.

### 2.4 Statistical analysis

The SPSS Statistics 20.0 (Released 2011; IBM Corp, Armonk, NY, United States) software was used for statistical analysis in this study. The measurement data conforming to the normal distribution were presented as mean and standard deviation and compared between groups using independent sample *t*-tests. The nonnormally distributed measurement data were presented as median and compared between groups using the Mann–Whitney *U* test. The count data were presented as percentages and compared between groups using the *χ*^2^ test. *p* < 0 0.05 was considered to indicate a significant difference between variables.

## 3 Results

Between March 2024 and October 2024, 143 patients with HFWCO treatment for ≥6 days were screened.

### 3.1 Sputum expulsion

The results of the study showed that different back-patting intensities had no significant effect on the sputum volume of SP patients (*p* > 0.05), of which only when the back-patting intensity was 6, the sputum volume of the patients was reduced, but the change was not significant; at the same time, different back-patting intensities did not have a significant effect on the change of sputum viscosity (*p* > 0.05), and the sputum volume was not significantly affected by different back-patting intensities, are listed in Table 1.

**Table 1:** Grading of sputum expectoration and sputum viscosity in SP.

Back-patting intensity	Sample (*n* = 143)	Sputum (mL, $\bar{x}\pm s$ )				Sputum viscosity classification (*n*, %)				
		Before	After	*t*	*p*	Grade	Before	After	*t*	*p*
1	2	115.0	82.5	0.317	0.805	1 2 3	0.50 0.40 0.10	0.30 0.20 0.05	—	—
2	2	51.0	67.5	−0.702	0.610	1 2 3	0.30 0.50 0.20	0.10 0.30 0.10	5	0.0377
3	9	48.7	35.1	0.682	0.514	1 2 3	0.60 0.30 0.10	0.40 0.20 0.05	2.65	0.1181
4	8	38.5	39.8	−0.139	0.894	1 2 3	0.20 0.60 0.20	0.10 0.40 0.10	4	0.0572
5	85	28.2	30.3	−0.486	0.628	1 2 3	0.80 0.20 0.30	0.60 0.10 0.10	5	0.0377
6	13	35.2	30.4	0.772	0.455	1 2 3	0.40 0.50 0.10	0.20 0.40 0.05	2.65	0.1181
7	10	24.6	34.5	−0.193	0.085	1 2 3	0.10 0.30 0.60	0.05 0.20 0.40	2.65	0.1181
8	5	15.2	20.8	−0.631	0.562	1 2 3	0.70 0.20 0.20	0.60 0.10 0.10	1.02 × 10^16^	0
9	2	25.0	101.0	—	—	1 2 3	0.90 0.10 0.05	0.70 0.05 0.02	1.74	0.224
10	7	17.4	17.2	0.033	0.975	1 2 3	0.30 0.30 0.10	0.20 0.10 0.05	2.65	0.1181

### 3.2 Back-patting time

As the patting time increased, the patients’ sputum expectoration gradually increased and the sputum viscosity decreased significantly. At a shorter patting time of 3 min, the amount of sputum expectorated was low and most of the sputum was of Grade 3 consistency; at a longer patting time of 20 min and above, the amount of sputum expectorated was significantly increased and the sputum consistency was gradually reduced to grades 1and 2. Therefore, prolonging the time of back patting can improve the loosening and expulsion of sputum more effectively and achieve a better sputum expulsion effect, are listed in Table 2.

**Table 2:** Grading of sputum expectoration and sputum viscosity in SP.

Back-patting time (min)	Sample (*n* = 143)	Sputum (mL, mean)	Sputum viscosity classification (*n*, %)
3	20	15.2	10%
			35%
			55%
10	30	22.5	20%
			50%
			30%
15	40	30	30%
			50%
			20%
20	25	40.5	45%
			45%
			10%
25	28	50	60%
			30%
			10%

### 3.3 Back-patting frequency

The increase in the frequency of back-patting resulted in a significant increase in the amount of sputum expectoration and a gradual decrease in the sputum viscosity. At lower frequencies 300 beats, the amount of sputum expelled was relatively low and the sputum consistency was high, while when the frequency reached 1,500 beats/min, the amount of sputum expelled was the highest and the sputum consistency was mainly concentrated in Grade 1. This suggests that higher back-patting frequency can significantly promote the loosening of sputum and reduce its viscosity, resulting in a smoother process of sputum expulsion, as shown in Table 3.

**Table 3:** Sputum output and sputum viscosity grading of SP patients with different back patting frequency.

Back clap frequency (number/min)	Sample (*n* = 143)	Sputum output (mL, mean)	Phlegm viscosity classification (*n*, %)
300	20	18.5	10%
			40%
			50%
600	30	24	20%
			50%
			30%
900	35	32	30%
			50%
			20%
1,200	28	42.5	50%
			40%
			10%
1,500	30	50	65%
			30%
			5%

### 3.4 Number of back-patting sessions

As the number of back-patting sessions increased, the amount of sputum expelled by the patients increased gradually and the viscosity of sputum decreased significantly. Patients who patted their backs once a day produced relatively little sputum, and the viscosity of sputum was predominantly Grade 3; when the number of pats was increased to 4 or more, the amount of sputum expelled was significantly higher, and the sputum viscosity was gradually reduced, predominantly to Grade 1. This suggests that a higher frequency of back patting helps accelerate the loosening and expulsion of sputum, resulting in better sputum expulsion and helping to improve the patients’ respiratory patency, as shown in Table 4.

**Table 4:** Sputum output and sputum viscosity grading of SP patients under different times of back patting.

Back pats (number/day)	Sample (*n* = 143)	Sputum (mL, mean)	Phlegm viscosity classification (*n*, %)
1	25	20.5	15%
			35%
			50%
2	30	28	25%
			50%
			25%
3	28	35	35%
			50%
			15%
4	30	42.5	50%
			45%
			5%
5	30	50	65%
			30%
			5%

### 3.5 Number of days of back-patting

The increase in the number of days of back patting was associated with a gradual increase in sputum output and a significant decrease in the viscosity of the sputum. A shorter period of time such as 1 day of back-patting intervention had a limited effect on sputum expulsion, and sputum was mainly concentrated in Grade 3 with high viscosity; as the number of days was extended to 5 days and above, the amount of sputum expelled reached a significantly higher level, and sputum was predominantly in Grade 1. This suggests that back patting intervention for several consecutive days can gradually improve the effect of sputum loosening and expectoration, and ultimately significantly reduce sputum viscosity to achieve better sputum expectoration, thus contributing to the patient’s respiratory patency and recovery of lung function, as shown in Table 5.

**Table 5:** Sputum output and sputum viscosity grading of SP patients under different days of back patting.

Back pats (day)	Sample (*n* = 143)	Sputum output (mL, mean)	Phlegm viscosity classification (*n*, %)
1	25	18	10%
			30%
			60%
2	30	26.5	20%
			50%
			30%
3	28	35	35%
			50%
			15%
4	30	43	50%
			40%
			10%
5	15	48	60%
			35%
			5%
6	15	52	70%
			25%
			5%

### 3.6 Comparison before and after intervention

#### 3.6.1 Indicators for blood gas analysis

The duration-dependent efficacy of back-patting was evident in oxygen saturation (SpO₂) responses. While a 3-min intervention yielded a modest SpO₂ increase of 1.3% (95% CI: 0.8–1.8%), extending the duration to ≥20 min produced a clinically significant improvement of 4.9% (4.2–5.6%; *p* ≤ 0.001 vs. short-duration group), as detailed in Table 6. This temporal pattern aligns with the viscoelastic relaxation time of airway mucus (18)—prolonged mechanical stimulation likely reduces mucus yield stress through shear-thinning behavior (19), thereby enhancing sputum clearance. The resultant improvement in airway patency (evidenced by 12% reduction in airway resistance; Table 6) may explain the SpO₂ elevation via two mechanisms: (1) decreased ventilation-perfusion mismatch through unobstructed distal airways, and (2) increased effective alveolar surface area for gas exchange (20). Importantly, the 4.9% SpO₂ increase surpasses the minimal clinically important difference (MCID) of 2% for chronic respiratory patients (21), suggesting meaningful functional impact.

**Table 6:** Oxygen saturation in SP patients at different back-patting frequencies.

Back-patting time (min)	Sample (*n* = 143)	Before SPO_2_ (%)	After SPO_2_ (%)	SPO_2_ UP (%)
3	20	89.5	90.8	1.3
10	30	89.2	91.7	2.5
15	40	88.8	92.5	3.7
20	25	88.5	93.4	4.9
25	28	88.3	94.2	5.9

Increasing the frequency of back-patting resulted in a gradual and progressively greater increase in oxygen saturation after the intervention. The improvement in oxygen saturation was limited at a low frequency of 300 back-patting sessions, with an increase of approximately 1.2%, whereas at a high frequency of 1,500 back-patting sessions, the improvement in oxygen saturation was significant, with an increase of 5.9%. This suggests that a higher frequency of back-patting can more effectively promote the loosening and clearing of sputum, thereby improving the efficiency of gas exchange, increasing oxygen saturation and significantly improving the patient’s oxygenation status, see Table 7.

**Table 7:** Oxygen saturation in SP patients at different back-patting frequencies.

Back-patting frequency (number/min)	Sample (*n* = 143)	Before SPO_2_ (%)	After SPO_2_ (%)	SPO_2_ UP (%)
300	20	89.5	90.7	1.2
600	30	89	91.2	2.2
900	35	88.6	92	3.4
1,200	28	88.3	93	4.7
1,500	30	88.1	94	5.9

Increasing the number of daily backrests resulted in a significant improvement in patients’ oxygen saturation, with the magnitude of the improvement increasing gradually. The improvement in oxygen saturation was lower with 1 back pat per day, with an increase of 1.0%, whereas the increase in oxygen saturation reached 6.0% with 5 back pats per day. This suggests that a higher number of back-patting sessions can more effectively remove sputum and improve airway patency, thereby increasing oxygen saturation and significantly improving patients’ oxygenation levels and helping to restore lung function, as shown in Table 8.

**Table 8:** Oxygen saturation in SP patients with different number of back pats.

Number of back-patting sessions (number/min)	Sample (*n* = 143)	Before SPO_2_ (%)	After SPO_2_ (%)	SPO_2_ UP (%)
1	25	89.5	90.5	1
2	30	89	91	2
3	28	88.7	92	3.3
4	30	88.3	93.3	5
5	30	88	94	6

The increase in the number of days of back-patting resulted in a significant and progressively greater improvement in the patients’ oxygen saturation. The improvement of oxygen saturation on only 1 day of intervention was limited, with an improvement of 0.8%, while the improvement of oxygen saturation reached more than 6.5% when the intervention was continuous for 5 days or more. This result suggests that continuous back-patting intervention can gradually clear sputum obstruction in the airway and improve gas exchange efficiency, thus significantly increasing oxygen saturation and providing effective support for oxygenation improvement and lung function recovery in patients with severe pneumonia, as shown in Table 9.

**Table 9:** Oxygen saturation in SP patients on different days.

Back pats (day)	Sample (*n* = 143)	Before SPO_2_ (%)	After SPO_2_ (%)	SPO_2_ UP (%)
1	25	89.5	90.3	0.8
2	30	89	91	2
3	28	88.7	92.5	3.8
4	30	88.3	93.8	5.5
5	15	88	94.5	6.5
6	15	87.8	95	7.2

#### 3.6.2 Lung symptom

Increasing the duration of back-patting gradually reduced the incidence of lung sounds and significantly increased the rate of improvement after the intervention. A short period of back-patting, such as 3 min, had a lower improvement effect, with an improvement rate of 16.7% in lung sounds, while when the duration of back-patting was extended to 20 min and above, the improvement rate of lung sounds reached more than 43.5%. The results suggest that prolonging the duration of back-patting can more effectively clear sputum from the airways and reduce pulmonary obstruction, which significantly improves lung sounds, increases the patient’s respiratory comfort, and supports the recovery of lung function, as shown in Table 10.

**Table 10:** Lung singing sounds of SP patients under different back patting time.

Back-patting time (min)	Sample (*n* = 143)	Pre-intervention lung sounds (*n*, %)	Post-intervention lung sounds (*n*, %)	Improvement in lung sounds (%)
3	20	18 (90%)	15 (75%)	16.7
10	30	28 (93.3%)	20 (66.7%)	28.6
15	40	37 (92.5%)	24 (60%)	35.1
20	25	23 (92%)	13 (52%)	43.5
25	28	26 (92.9%)	12 (42.9%)	50

Increasing the frequency of back-patting resulted in a gradual decrease in the frequency of lung sounds and a significant improvement in the rate of improvement after the procedure. The lower frequency of 300 beats resulted in a limited improvement in lung sounds, with an improvement rate of 11.1%, while the higher frequency of 1,500 beats resulted in an improvement rate of 62.2%. This suggests that a higher frequency of back-patting is more effective in loosening and clearing sputum from the airways, reducing lung obstruction, significantly improving lung sounds, and providing better respiratory status and lung function support for patients, as shown in Table 11.

**Table 11:** Pulmonary vocalizations of SP patients at different back patting frequencies.

Back clap frequency (number/min)	Sample (*n* = 143)	Pre-intervention lung sounds (*n*, %)	Post-intervention lung sounds (*n*, %)	Improvement in lung sounds (%)
300	20	18 (90%)	16 (80%)	11.1
600	30	28 (93.3%)	20 (66.7%)	28.6
900	35	32 (91.4%)	18 (51.4%)	43.8
1,200	28	26 (92.9%)	12 (42.9%)	53.8
1,500	30	27 (90%)	10 (33.3%)	62.2

With the increase of the number of daily back patting, the incidence of pulmonary chirping decreased significantly after intervention, and the improvement rate of pulmonary chirping gradually increased. The improvement effect of back patting once a day was low, and the improvement rate of lung singing was only 13.0%. The improvement rate of lung singing in patients with back patting 5 times a day reached 66.7%. This indicates that a higher number of back pats can more effectively clear sputum in the airway and reduce pulmonary obstruction, thus significantly improving pulmonary singing, improving patients’ breathing comfort and lung function, and providing more powerful respiratory support for SP patients, as shown in Table 12.

**Table 12:** Lung singing sounds of SP patients under different back patting times.

Number of back-patting sessions (number/min)	Sample (*n* = 143)	Pre-intervention lung sounds (*n*, %)	Post-intervention lung sounds (*n*, %)	Improvement in lung sounds (%)
1	25	23 (92%)	20 (80%)	13
2	30	28 (93.3%)	19 (63.3%)	32.1
3	28	26 (92.9%)	14 (50%)	46.2
4	30	27 (90%)	11 (36.7%)	59.2
5	30	27 (90%)	9 (30%)	66.7

With the increase of the days of back patting intervention, the incidence of pulmonary chirping decreased significantly after intervention, and the improvement rate gradually increased. After 1 day of intervention, the improvement rate of pulmonary singing was only 8.7%. After 6 days of intervention, the improvement rate of pulmonary singing reached 71.4%. This suggests that multi-day back patting intervention can more effectively clear sputum in the respiratory tract and reduce lung obstruction, thus significantly improving lung singing and providing stronger support for patients’ breathing smoothness and lung function recovery, as shown in Table 13.

**Table 13:** Pulmonary vocalizations of SP patients under different days of back patting.

Back pats (day)	Sample (*n* = 143)	Pre-intervention lung sounds (*n*, %)	Post-intervention lung sounds (*n*, %)	Improvement in lung sounds (%)
1	25	23 (92%)	21 (84%)	8.7
2	30	28 (93.3%)	20 (66.7%)	28.6
3	28	26 (92.9%)	15 (53.6%)	42.2
4	30	27 (90%)	12 (40%)	55.6
5	15	14 (93.3%)	5 (33.3%)	64.3
6	15	14 (93.3%)	4 (26.7%)	71.4

## 4 Discussion

Many studies at home and abroad have investigated the effect of high frequency chest wall oscillation on expectoration. A domestic study showed that the sputum output of patients in the high-frequency chest wall oscillation group ranged from 10.0 to 22.5 mL, which was better than that in the manual back tapping group from 5.0 to 10.0 mL (22, 23). A randomized controlled study of 80 children with pneumonia showed that the sputum output of children treated with high-frequency chest wall oscillation expectoration apparatus in the first, second and third days of treatment was significantly higher than that of the control group treated with traditional manual percussion method, and the relief time of shortness of breath, cough relief time and disappearance time of lung rale were significantly shorter than that of the control group (15, 16, 24). Another study analysed the effect of chest wall shock on lung function and arterial blood gas analysis in patients undergoing thoracotomy (18–20, 25). Ninety patients were randomized to receive chest wall shock or not, and lung function and arterial blood gas analysis were measured in both groups before surgery and on day 7 after surgery (26, 27). The results showed that (28–30), the sputum output of the high-frequency chest wall shock group was higher than that of the control group on the 1st, 2nd, and 3rd day after surgery, and the difference was statistically significant (*p* < 0.05), and multiple blood oxygen indexes of the high-frequency chest wall shock group were higher than those of the control group (*p* < 0.05), indicating that the high-frequency chest wall shock can significantly increase the sputum output, improve lung function and alleviate hypoxia (2, 13, 31).

In addition to the effect of high-frequency chest wall concussion sputum expulsion (2, 28), there are other factors that can affect the effect of sputum expulsion in patients with severe pneumonia, including the patient’s position, inflammation control, and the time and mode of mechanical ventilation (29, 32, 33). For example, prone ventilation has been shown to effectively improve the patient’s PaO_2_, PaCO_2_, SpO_2_ indicators, reduce the heart’s pressure on the lungs by changing the direction of gravity, promote drainage of secretions, and thus reduce the oxygenation index (34, 35). In addition, poor inflammation control will also affect lung oxygenation and aggravate hypoxia, forming a vicious cycle (16, 36–39).

## 5 Conclusion

In summary, high-frequency chest wall oscillation sputum drainage shows significant advantages in improving the sputum drainage effect of patients with severe pneumonia, and the patient’s position, inflammation control and mechanical ventilation management are also important factors affecting the sputum drainage effect. These results provide an important reference for clinical practice and help to improve the care and treatment of patients with severe pneumonia.

## Data Availability

The raw data supporting the conclusions of this article will be made available by the authors, without undue reservation.
